# Stable Logic Operation of Fiber-Based Single-Walled Carbon Nanotube Transistor Circuits Toward Thread-Like CMOS Circuitry

**DOI:** 10.3390/ma11101878

**Published:** 2018-10-01

**Authors:** Jae Sang Heo, Kyung-Tae Kim, Seok-Gyu Ban, Yoon-Jeong Kim, Daesik Kim, Taehoon Kim, Yongtaek Hong, In-Soo Kim, Sung Kyu Park

**Affiliations:** 1 School of Electrical and Electronic Engineering, Chung-Ang University, Seoul 06980, Korea; heojs38@gmail.com (J.S.H.); ktkim0314@gmail.com (K.-T.K.); skban39@naver.com (S.-G.B.); kyj9122875@naver.com (Y.-J.K.); 2 Department of Medicine, University of Connecticut Health Center, Farmington, CT 06030, USA; ikim@uchc.edu; 3 Department of Electrical and Computer Engineering, Seoul National University, Seoul 08826, Korea; goodkds@snu.ac.kr (D.K.); rhlight4@snu.ac.kr (T.K.); yongtaek@snu.ac.kr (Y.H.)

**Keywords:** fiber-based electronics, single-walled carbon nanotubes (SWCNTs), thin-film-transistors (TFTs), logic circuits, e-textile

## Abstract

A fiber-based single-walled carbon nanotube (SWCNT) thin-film-transistor (TFT) has been proposed. We designed complementary SWCNT TFT circuit based on SPICE simulations, with device parameters extracted from the fabricated fiber-based SWCNT TFTs, such as threshold voltage, contact resistance, and off-/gate-leakage current. We fabricated the SWCNTs CMOS inverter circuits using the selective passivation and n-doping processes on a fiber substrate. By comparing the simulation and experimental results, we could enhance the circuit’s performance by tuning the threshold voltage between p-type and n-type TFTs, reducing the source/drain contact resistance and off current level, and maintaining a low output capacitance of the TFTs. Importantly, it was found that the voltage gain, output swing range, and frequency response of the fiber-based inverter circuits can be dramatically improved.

## 1. Introduction

Recently, portable, wearable, patchable, and implantable systems have required high performance and flexible electronics. Flexible electronics, such as flexible printed circuit board (PCB)-based and polydimethylsiloxane (PDMS)-based electronics, are used for a variety of wearable electronic applications [1,2,3]. Also, electronic textiles (e-textiles) have been highlighted as the next generation of smart and wearable electronics for human-friendly applications, because they are light weight, have deformability in movement, and have a wide range of applications within various fields [4,5]. To use e-textile devices in real life applications, there are several major obstacles to overcome. Firstly, e-textile device fabrication technologies should be easily adaptable to existing textile processes for low cost manufacturing. Secondly, a high degree of mechanical flexibility/durability and washability must be achieved for long-term use. Lastly, high performance active devices, such as thin-film-transistors (TFTs) and light-emitting diodes (LEDs), should be developed for electronic circuit operations including fast-switching, amplification, and digital logics.

Over the last few decades, a number of researchers have reported on e-textile fabrication processes of fiber-based TFTs [6]; LEDs [7,8]; and energy harvesting and storage devices [9,10,11] using polymer, inorganic, and organic materials. Because of these advances, highly flexible and comfortable e-textile devices are widely used in a broad range of emerging applications [12]. In addition, e-textiles could be used for various adaptable formats such as electronic apparel and body patchable-type devices, leading to significant impacts on the development of wearable electronics. Recently, research groups have demonstrated a one-dimensional (1D) n-type metal oxide field effect transistor-based inverter circuit using an external load resistor [13], and a single-walled carbon nanotube (SWCNT)-based complementary CMOS integrated circuit on a cylindrical-shaped fiber substrate using a simple selective doping method [12]. Although the reported results are noteworthy as building blocks for utilizing e-textiles, the circuit integration of the devices and the demonstration of logic circuit operations with fiber-based SWCNT TFTs are still challenging. This limitation greatly prohibits the use of e-textile devices in more practical applications, such as fully weaved e-textiles and integrated multi-functional e-textile systems.

In this study, we report the design of effective fiber-based TFT circuits showing rail-to-rail operation and high frequency responses. To do this, we have modeled fiber-based SWCNTs and have optimized the circuit designs using an Automatic Integrated Circuit Modeling (AIM)-SPICE simulation. Furthermore, we fabricated integrated circuits and demonstrated the logic inverter operation. We assumed that the bottom gate bottom contact (BGBC) structure and the large cross-over area between the unpatterned gate and the source/drain electrodes, caused by fiber-based device configurations, can lead to a high contact resistance and large off-/gate-leakage current, which results in limited rail-to-rail operations and poor frequency responses [14,15]. In addition, we proposed that threshold voltage and n-type doping concentration significantly affects the gain of fiber-based SWCNTs CMOS logic circuits [16]. From the characteristics of the demonstrated CMOS logic circuits and the simulation results using AIM-SPICE, we could design a small gate to source/drain cross-over area, low source/drain contact resistance, and accurate tuning of the threshold voltage for each of the TFTs. Consequently, we could mitigate the unideal static and dynamic operation of the fiber-based CMOS logic circuitry.

The rest of this article is organized as follows: Section 2 describes the fabrication process of SWCNT TFT and complementary TFT integrated circuits in detail, and Section 3 shows both the simulation and experimental results of the SWCNT TFTs and circuit operations. Section 4 summarizes the results of this study and conclusions are described.

## 2. Experimental Procedure

**Figure 1:**
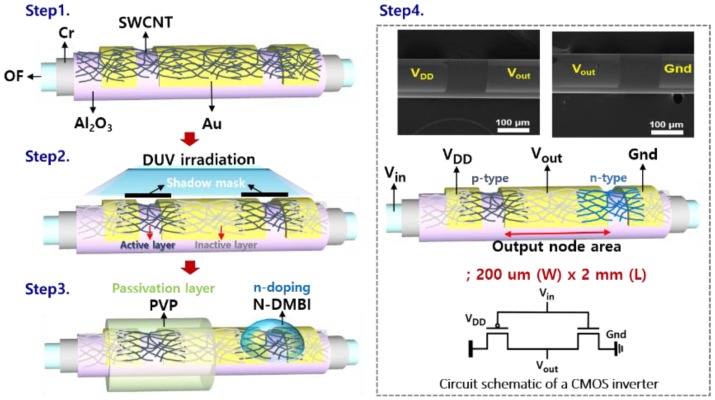
Schematic of fabrication process and scanning electron microscope (SEM) images of the fiber-based SWCNTs (single-walled carbon nanotubes) CMOS inverter circuit.

For the demonstration of a CMOS inverter circuit on a cylindrical fiber substrate, optical fibers (OFs) (diameter 125 µm) and SWCNTs were used as the substrate and active layers, respectively. The SWCNT solution was prepared by dissolving high-pressure CO (HiPCO) SWCNT and poly(3-doedcylthiophene) (P3DDT) in toluene (25 mL), at a weight ratio of 1:1.25, followed by ultra-sonication (Ultrasonic processors, 400 W, Branson Sonifier 450, Branson, MO, USA) for 30 min in a cooling bath, and centrifugation for 1 h at 10,000 rpm. In addition, for the electrical characterizations of the fiber-based SWCNTs TFTs, a semiconductor parameter analyzer (Agilent 4156C, Agilent Technologies, Santa Clara, CA, United States) was used under ambient conditions, and the saturation field-effect mobility (*μ_sat_*) was defined using the following equation:

$$I_{\mathrm{DS}}=\frac{WC_{i}}{2L}\mu_{\mathrm{sat}}{\left(V_{\mathrm{GS}}-V_{\mathrm{TH}}\right)}^{2}$$

where *W* (196.25 µm) and *L* (125 µm) are channel width and length, respectively; *C_i_* is the capacitance of the gate dielectric; and *μ_sat_* is the saturation field-effect mobility. As shown in Figure 1, for the bottom gate and bottom contact structured TFTs, a Cr (88 nm) layer was formed on an OF substrate using radio frequency magnetron sputtering, and an A_2_O_3_ (30 nm) layer was deposited on the Cr layer as a gate insulation layer using the atomic-layer-deposition (ALD) (150 °C) process. For the source/drain electrode contact, Au was deposited on the Al_2_O_3_ layer by a thermal evaporation process with a shadow mask. Subsequently, the SWCNTs were coated with a partial reel-process and were patterned using a spatial deep ultraviolet (DUV) irradiation, with wavelengths of 254 nm and 185 nm [12]. For the selective passivation and n-doping of the SWCNTs, half of the OF substrate was submerged in a poly(4-vinylphenol) (PVP) solution for 1 min, followed by annealing at 150 °C for 30 min. Then, the n-type dopant, (4-(1,3-dimethyl-2,3-dihydro-1*H*-benzoimidazol-2-yl)-phenyl)-dimethylamine (N-DMBI) (0.5 wt %), was coated on the uncovered channel region by drop-casting, and was annealed at 80 °C for 1 h in a N_2_ atmosphere [12,16]. Lastly, the SWCNTs CMOS inverter circuit was successfully demonstrated on a single fiber substrate (Figure 1, Step 4). The SWCNT TFT device model parameters have been extracted for the AIM-SPICE level 15 model [17]. Then, the transfer (I_DS_−V_GS_) and output (I_DS_−V_DS_) curves were obtained using the AIM-SPICE simulation, as well as the device characteristics for the stable (rail-to-rail) operation of the CMOS circuits, including the threshold voltage, off current, contact resistance, and the size of the source/drain contact node.

## 3. Results and Discussion

**Figure 2:**
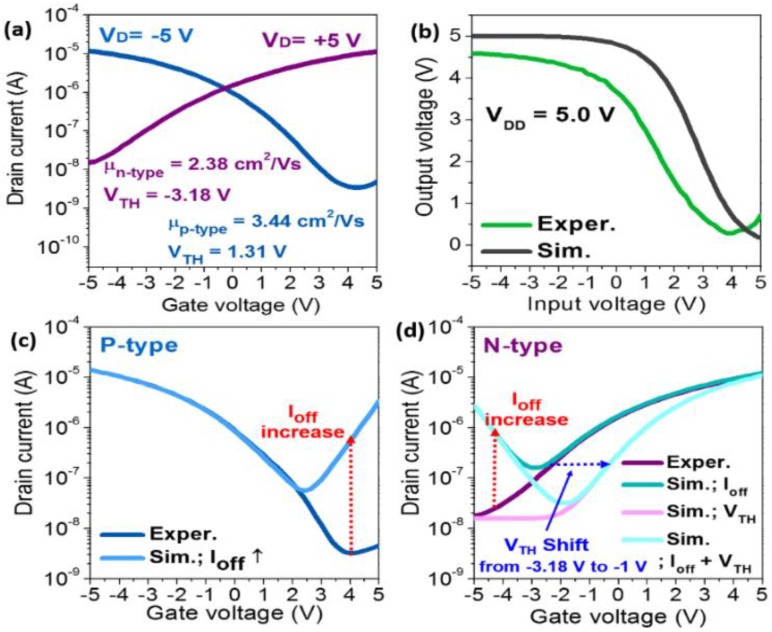
(**a**) Transfer curves of p-type and n-type of fiber-based SWCNTs transistors; (**b**) the experimental (Exper.) and the simulated (Sim.) voltage transfer curves of the fiber-based CMOS inverter circuits; and (**c**) the p-type and (**d**) n-type fiber-based SWCNT transistors when applying several variable parameters.

As shown in Figure 2a, n-type and p-type fiber-based TFTs show saturation mobilities of 2.38 cm^2^ V^−1^ s^−1^ and 3.44 cm^2^ V^−1^ s^−1^, respectively. The average field-effect mobilities are 1.62 cm^2^ V^−1^ s^−1^ and 3.61 cm^2^ V^−1^ s^−1^ for n- and p-type SWCNTs TFTs, respectively (with a standard deviation of 0.63 cm^2^ V^−1^ s^−1^ and 0.3 cm^2^ V^−1^ s^−1^, respectively, from 16 devices in the same batch). These results show that our fiber-based devices have a high uniformity. In addition, for the realization of CMOS circuits with SWCNTs transistors on a fiber substrate, n-doping and passivation processes were implemented in order to be selectively converted into n-type TFTs using N-DMBI and PVP, respectively. The fiber-based CMOS inverter exhibited a voltage gain of 3.37 V/V at a V_DD_ of 5.0 V, as shown in Figure 2b, which is comparable to planar-type inverter circuits using SWCNTs [18]. Although the individual TFTs and inverter circuits have shown a relatively good performance, the inverter circuit often exhibits incomplete rail-to-rail operations (Figure 2a). To overcome this issue, we optimized the circuit design using an AIM-SPICE simulation with TFT device parameters and CMOS inverter operational characteristics. In fact, for the rail-to-rail operations with high gain of logic inverter circuits, several parameters should be considered, such as the threshold voltage (V_TH_) tuning of the switching devices, a low off-current (I_off_) with minimal gate leakage, a low source/drain contact resistance (Rc), and so forth. In our fiber-based SWCNTs CMOS logic circuits, a large overlap area between the unpatterned gate electrode and the source/drain electrodes, and the bottom contact device configuration can lead to a large I_off_, including a high gate leakage and high source/drain contact resistance (Rc) (300 kΩ), respectively, which is inevitable because of the nature of the CMOS fabrication process. We have provided the values of the contact resistance (Rc) extracted using the transmission line method (TLM) reported in our previous study [12]. For the extraction of the contact resistance, the total resistance (R_T_) was plotted as a function of the channel length, where the total resistance is given by R_T_ = R_CH_ + 2 Rc (R_CH_—channel resistance). In addition to the structural issues, in some case, such as for a too low or too high N-DMBI doping concentration, large I_off_ and V_TH_ variations were frequently observed in the negative or positive V_GS_ region in both n- and p-type TFTs, which is similar to the ambipolar transport behaviors. Therefore, systematic device-level circuit modeling was carried out, using the experimental data and the extracted device parameters to gain more insight into the aforementioned behaviors. For the simulation modeling, we considered several variable parameters such as I_off_, Rc, and V_TH_ (calculated from the square-root of I_D_, not shown here), which were extracted from the experimental data on the SWCNTs TFTs (Figure 2a), and we then applied these values to the AIM-SPICE simulation (Figure 2b). As shown in Figure 2c,d, thep- and n-type SWCNTs TFTs with an I_off_ increase and V_TH_ shift (toward positive voltage, for n-type) were simulated to mimic the uncontrollably doped p- and n-type SWCNTs TFTs. In addition, we set the V_TH_ to 1.0 V for the p-type TFTs and for the variables of the n-type TFTs, because the V_TH_ for the p-type TFT was typically less dependent on the processing conditions compared with the n-type TFTs.

**Figure 3:**
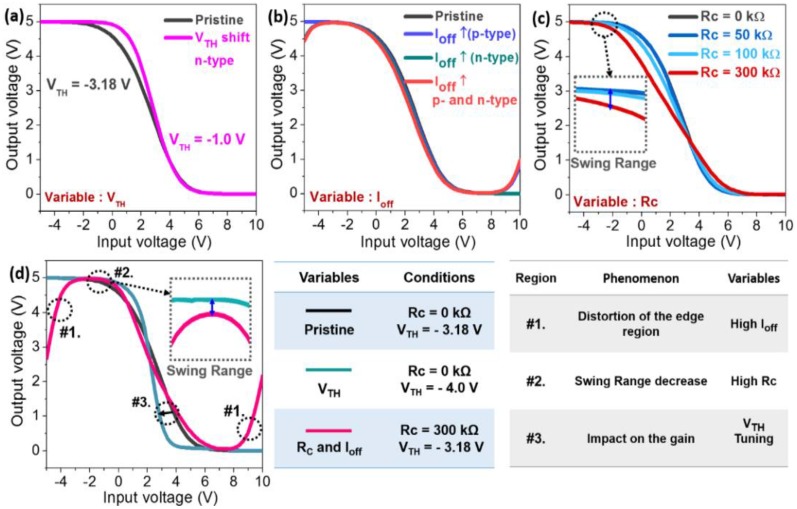
The simulated voltage transfers of the fiber-based CMOS inverters, depending on various parameters, with (**a**) a threshold voltage (V_TH_) (only n-type); (**b**) low off-current (I_off_); (**c**) contact resistance (Rc); and (**d**) a high I_off_ and Rc (300 kΩ), as well as a summary of the influences on the fiber-based CMOS inverters, depending on several variable parameters.

In addition to the simulation data for the unit TFT devices, we have modeled fiber-based CMOS inverters using several variable parameters that were extracted from the simulated SWCNT transistors, as shown in Figure 3. As can be seen in Figure 3a, the tuning of V_TH_ between the p- and n-type TFTs can have a significant impact on the gain (slope) of the inverter, as is expected. Additionally, the increasing I_off_ of the n- and p-type SWCNT transistors (ambipolar behaviors) deteriorates the edge region of the voltage transfer curves, which, in turn, is caused by the deteriorating driving force of the inverter circuits (Figure 3b). However, although the tuning of V_TH_ and the decreasing I_off_ can improve the voltage transfer characteristics of fiber-based CMOS inverters, the inverters cannot show the complete rail-to-rail operation because of a high source/drain Rc. As shown in Figure 3c, the Rc can have serious effects on the gain and swing range, resulting in unconventional rail-to-rail characteristics of the CMOS inverter. In Figure 3d, it is noteworthy that the logical rail-to-rail operations, particularly, the flat edge region (#1) and the large swing range (#2) of the fiber-based SWCNTs CMOS inverters, are strongly dependent on the low off-current and minimal contact resistance of the unit TFT devices, respectively. Furthermore, the V_TH_ tuning between the p- and n-type switching devices seems to have a significant impact on the gain of the inverter circuits (#3). Similar experimental results for planar-type SWCNTs CMOS inverter circuits were also reported previously [19,20].

**Figure 4:**
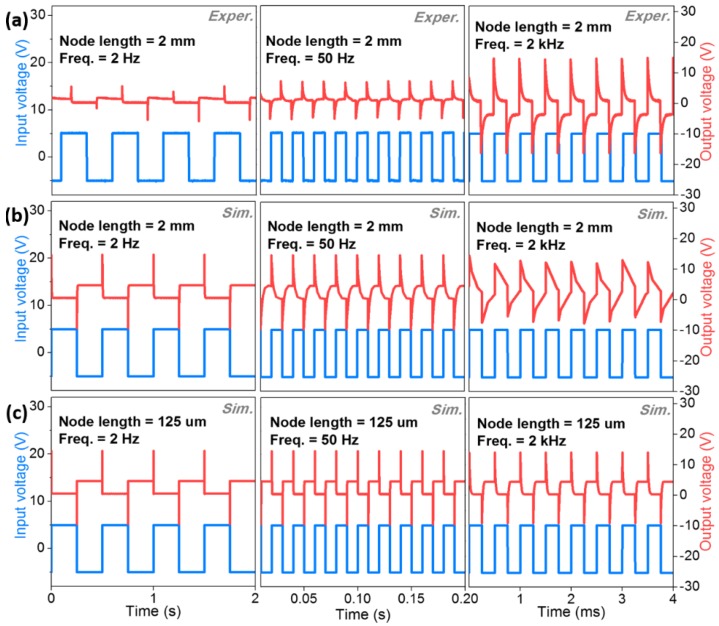
The dynamic response characteristics of fiber-based CMOS inverter circuits with a large output node length (2 mm) based on (**a**) experimental and (**b**) simulated results; and (**c**) a small output node length (125 µm) based on simulated results.

Generally, in the case of dynamic circuit operations, the resistor–capacitor (RC) time constant can be typically considered as one of the most important factors. Therefore, in order to achieve a high frequency response and to suppress the unexpected overshooting oscillations for the inverter circuits, the parasitic capacitance and resistance related to the RC factor should be reduced [21,22]. To ensure the dynamic operation of the fiber-based CMOS circuits, we investigated the frequency response of the fiber-based CMOS inverter using device-level circuit modeling, which was performed by driving the input of that inverter from a pulse generator while monitoring the input and output signals with a two-channel oscilloscope. As we mentioned in the manuscript, as the fiber-based CMOS inverter circuits were fabricated with the bottom contact configuration and unpatterned gate and source/drain electrodes, a large overlapped capacitance was observed, particularly in the output node contact area of that circuit (node contact area, width 0.2 mm × length 2 mm). Therefore, we can expect that a very large RC-induced propagation delay, which is attributed to the extremely high output capacitance and may dominate the frequency response properties of the fiber-based CMOS inverter circuits. Nevertheless, Figure 4a indicates the experimentally obtained results, which are of a 20%~50% performance compared to the modeling results. Although the output signal is notably distorted at this frequency, possibly due to the large RC-induced propagation delay, the fiber-based CMOS inverters are indeed able to follow the 50 Hz input signal. In the case of a further high input frequency of 2 kHz, the circuits, in fact, lose their logic properties, demonstrating critically distorted and illogical behaviors for both the experimental and simulated results (Figure 4a,b). On the other hand, the frequency response characteristics of the fiber-based CMOS inverter circuits were significantly improved as output-contact node area was scaled down to width of 200 µm and length of 125 µm, as shown in Figure 4c. Even at a frequency of 2 kHz, the frequency response showed that the output signal was able to follow the 2 kHz input signal. From this result, it can be seen that the small gate to source/drain overlap (output capacitance) area will be of great importance for future high-performance fiber-based circuits [22,23]. Consequently, in the case of the fiber-based SWCNTs CMOS inverter circuits, various factors, such as patterning processes for low I_off_, n-type doping processes for stable and reliable V_TH_, and the design of device structures for low Rc and RC, have to be carefully considered in order to achieve high-performance, complementary logic circuits on fiber-based substrates for practical applications.

## 4. Conclusions

In summary, we have demonstrated static and dynamic operations of one-dimensional fiber-based SWCNTs CMOS inverter circuits. On the basis of the experimental and simulation results, various parameters that determine the circuit performance were systematically investigated. The results show that the low off-current, minimal contact resistance, and threshold voltage tuning of unit fiber-based TFTs have a significant impact on the logical operation of the inverter circuits. Notably, the dynamic characteristics of inverter circuits could also be enhanced by reducing the overlapped output node area, which can lead to a large RC-induced propagation delay. Although the circuit performance could be improved by the appropriately sized n-type and p-type devices, their electrical performances are still unsatisfactory for full applications in consumer level e-textile systems. Consequently, high-performance alternative materials; their corresponding fabrication technologies; and careful circuit design, including the tuning of various parameters within the device levels, should be developed in order to achieve next generation e-textile systems with robust and high-performance circuits.
